# Multifocal Fibromuscular Dysplasia in a Horseshoe Kidney: Coincidence or Consequence?

**DOI:** 10.7759/cureus.95561

**Published:** 2025-10-28

**Authors:** Jasper Luijkx, Roy J Claessen, Guy J Mostard, Daan J Van Twist

**Affiliations:** 1 Department of Internal Medicine, Zuyderland Medical Centre, Sittard-Geleen, NLD

**Keywords:** cardiovascular disease, horseshoe kidneys, hypertension, renal fibromuscular dysplasia, spontaneous coronary artery dissection

## Abstract

Spontaneous coronary artery dissection (SCAD) is increasingly identified as a significant cause of acute coronary syndrome and is often associated with fibromuscular dysplasia (FMD), a non-atherosclerotic, non-inflammatory vascular disease affecting medium-sized arteries. SCAD and FMD frequently co-occur, warranting extracoronary vascular imaging. Horseshoe kidney, a rare congenital anomaly, has been linked to various genitourinary and vascular abnormalities but has not previously been associated with FMD. A man in his early 50s presented with acute chest pain and was diagnosed with SCAD of the obtuse marginal artery. Due to the known association between SCAD and FMD, CT angiography was performed, revealing a horseshoe kidney and a classic string-of-beads appearance in both renal arteries, consistent with multifocal FMD. No abnormalities were found in other vascular beds, and there was no clinical evidence of connective tissue disorders. Renal function was normal, and hypertension was well controlled with antihypertensive medication. The patient was managed conservatively and remains under outpatient follow-up. This is the first reported case of coexisting renal artery multifocal FMD and a horseshoe kidney. While the co-occurrence may initially appear coincidental, the rarity of both conditions suggests a potential, previously unrecognized congenital or biomechanical association. One could hypothesize that the abnormal positioning and limited mobility of the fused kidneys in a horseshoe kidney might contribute to chronic vascular wall stress and arterial remodeling. Further research is warranted to assess the prevalence of FMD in patients with horseshoe kidneys and to explore possible shared developmental or mechanical mechanisms.

## Introduction

Spontaneous coronary artery dissection (SCAD) is an increasingly recognized cause of acute coronary syndrome (ACS) [1]. SCAD may arise from a tear in the tunica intima, allowing blood from the lumen to enter the tunica media. Within the tunica media, the blood accumulates and forms a hematoma in the arterial wall (intramural hematoma) [2]. Furthermore, SCAD can also result from disruption of the vasa vasorum, microvessels that supply blood to the arterial wall itself, leading to leakage of blood into the tunica media, resulting in an intramural hematoma. In both cases, the hematoma may spread longitudinally within the tunica media, compressing the coronary lumen over a long distance and causing ACS.

In many patients with SCAD, both the affected and unaffected coronary arteries exhibit increased tortuosity [3]. This winding or curviness suggests a pre-existing abnormality of the arterial wall. Indeed, in the majority of cases, SCAD is associated with a systemic vasculopathy [1,2]. SCAD may occur in connective tissue disorders such as Ehlers-Danlos syndrome. However, multifocal fibromuscular dysplasia (FMD) is the most commonly found vasculopathy, with reported prevalence rates of up to 77% [1]. In that case, typical string-of-beads lesions, which are segments with alternating areas of dilatation and stenosis, can be observed in extracoronary medium-sized arteries, including the renal, carotid, iliac, and visceral arteries [4]. Additionally, aneurysms and increased tortuosity may also be present [4,5]. Given the association between SCAD and FMD, it is recommended to screen all patients with SCAD for the presence of multifocal FMD in extracoronary arteries by performing imaging studies (usually CT angiography, CTA) from the brain to the pelvis [1,4].

We recently encountered a unique case of a patient with SCAD who was found to have multifocal renal artery FMD but who was also diagnosed with a horseshoe kidney: a congenital condition in which both kidneys are fused at the lower poles, forming a typical U or horseshoe shape [6]. To the best of our knowledge, the combination of SCAD/FMD and a horseshoe kidney has not been previously reported. As both conditions are rare, this raises the intriguing question of whether this co-occurrence is purely coincidental or suggests a previously unrecognized association.

## Case presentation

A man in his early 50s was admitted to the ED with acute chest pain. His medical history revealed hypertension, diagnosed in his mid-40s, which reportedly improved with lifestyle modifications. There was no history of migraines or pulsatile tinnitus. The pain began during the night and was accompanied by a tingling sensation in both arms and sweating.

In the morning, the patient consulted his general practitioner. Electrocardiography showed no ST-segment elevations. However, as troponin-T levels were elevated in a blood sample obtained by the general practitioner eight hours after symptom onset (23 ng/L; reference range: <14 ng/L for the assay used), he was referred to the ED. As subsequent troponin-T levels increased by more than 20% (28 ng/L and 38 ng/L in samples obtained 12 and 26 hours after symptom onset, respectively), a diagnosis of non-ST-elevation ACS was established.

**Figure 1:**
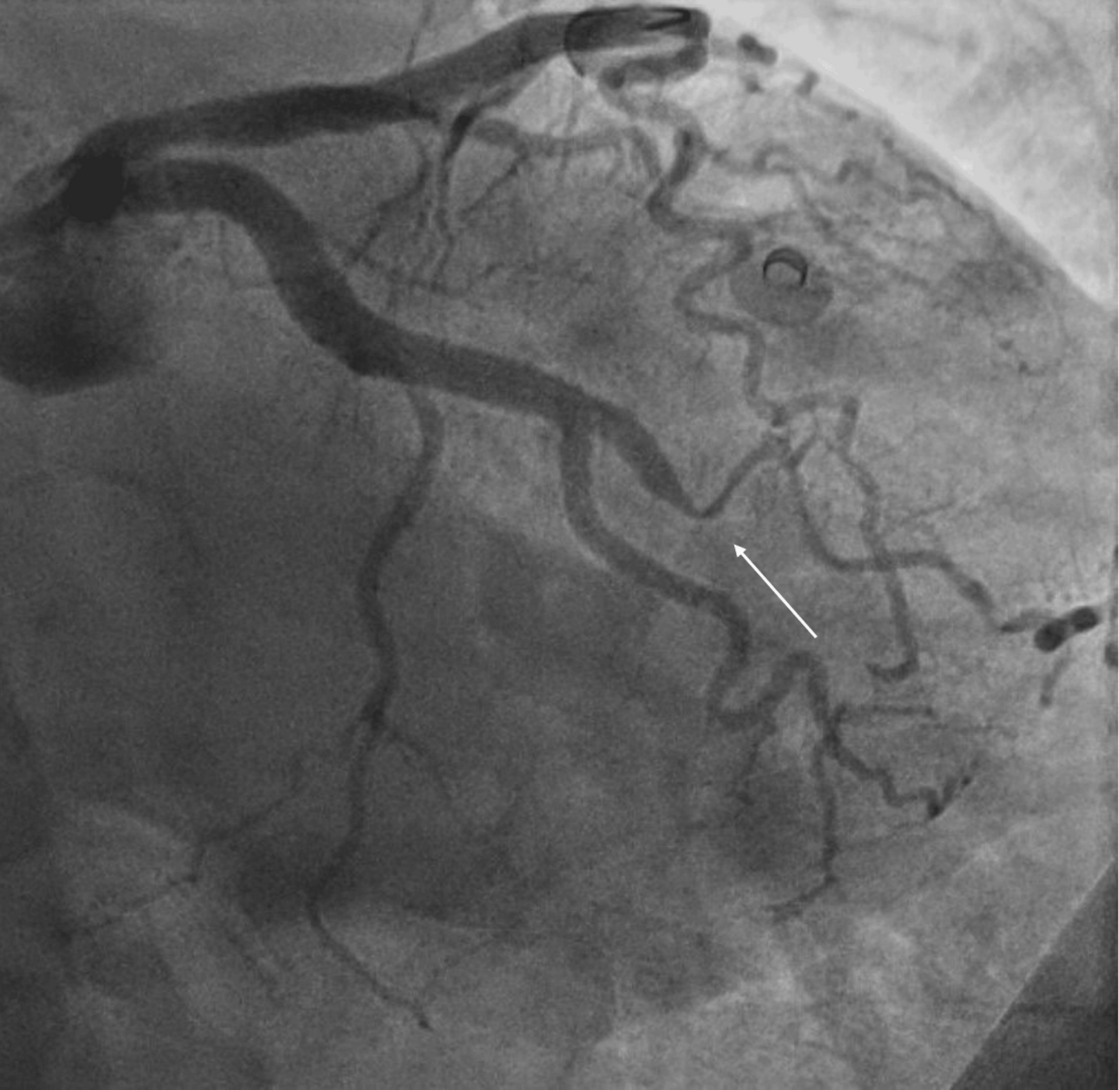
Coronary angiography Coronary angiography showing diffuse and smooth narrowing of the middle segment of the obtuse marginal artery (arrow), consistent with a type 2a SCAD. Moreover, the coronary tortuosity of the other coronary arteries is noted. SCAD, spontaneous coronary artery dissection

Coronary artery angiography revealed diffuse, smooth narrowing of the middle segment of the obtuse marginal artery. There was no evidence of atherosclerotic plaques, such as focal stenoses, calcifications, or irregular vessel wall contours. The absence of these features, combined with the characteristic angiographic appearance of type 2a SCAD, characterized by long, smooth, tapered narrowing, led to the diagnosis of SCAD (Figure 1).

According to the prevailing recommendations at the time [1], conservative management with dual antiplatelet therapy was initiated. As blood pressure was elevated during hospital admission, perindopril was initiated.

**Figure 2:**
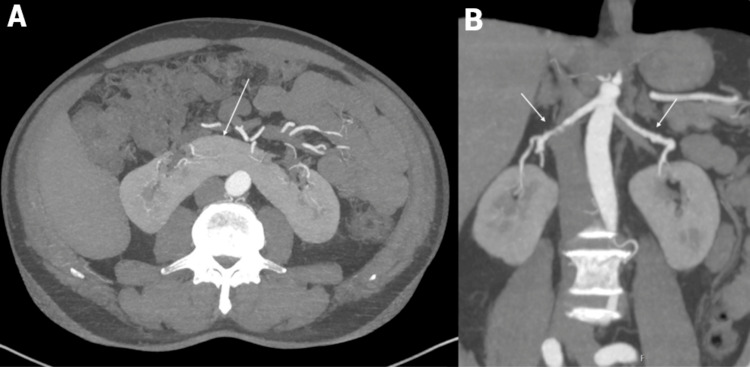
CT scan Contrast-enhanced CTA of the abdomen. (A) Axial reconstruction revealing a horseshoe kidney with fusion of the lower poles of both kidneys in the midline (arrow). (B) Coronal reconstruction with a typical string-of-beads appearance of both renal arteries (arrows), which is characteristic of multifocal FMD. CTA, CT angiography; FMD, fibromuscular dysplasia

Given the association of SCAD with extracoronary vascular abnormalities, a CTA was performed. This revealed a horseshoe kidney (Figure 2A) with fusion of the lower poles of both kidneys. Additionally, the characteristic “string-of-beads” appearance, typical of multifocal FMD, was observed in both renal arteries on CTA reconstructions in the axial, sagittal, and coronal planes (Figure 2B).

CTA revealed no abnormalities in the visceral, carotid, or intracranial arteries. Moreover, there was no evidence of connective tissue disorders or syndromic abnormalities. As the patient reported no urinary symptoms, showed no evidence of kidney disease (estimated glomerular filtration rate > 90 ml/min/1.73 m², no hematuria or albuminuria), and had well-controlled blood pressure on perindopril, he was managed conservatively and will be followed in our outpatient clinic.

## Discussion

We report on a patient with a unique combination of SCAD, multifocal FMD, and a horseshoe kidney. To the best of our knowledge, multifocal FMD of the renal arteries has not previously been reported in patients with a horseshoe kidney. We conducted a PubMed search for publications available up to October 1, 2025, using the following keywords: (1) “horseshoe kidney” and (2) “fibromuscular dysplasia” or “spontaneous coronary artery dissection”, without language or article type restrictions. Additionally, we reviewed seminal publications on FMD and SCAD and screened their reference lists for any mention of horseshoe kidney. This search yielded only one report describing a solitary renal artery stenosis in a patient with a horseshoe kidney [7], but no reports of multifocal FMD.

According to the classical theory, a horseshoe kidney forms during the fourth week of gestation, when the kidneys remain close together in the pelvis and the renal capsule has not yet developed [6,8]. At this stage, the lower poles of the nephrogenic blastemas may come into contact and fuse at the midline, forming a horseshoe-shaped kidney. The horseshoe kidney remains malrotated because the kidneys cannot ascend to their normal position, as the fused isthmus is trapped beneath the inferior mesenteric artery. Arterial vascularization is often normal, with two renal arteries (left and right) branching into upper, middle, and lower segments. However, similar to normal kidneys, variations with two or three renal arteries on each side may also occur [6,8]. The isthmus can also be supplied by arteries arising below it, originating from the abdominal aorta or common iliac arteries. For a more detailed overview of variants in arterial vascularization in the horseshoe kidney, we refer to Natsis et al. [6].

Variations of the inferior vena cava are commonly observed in patients with a horseshoe kidney, including left-sided, pre-isthmic, or double vena cava [6,8]. Additionally, abnormalities of the urinary tract (including the calyces and ureters) may be present, possibly related to the aforementioned malrotated position of the kidney. Furthermore, horseshoe kidneys can be associated with chromosomal abnormalities such as trisomy 13, 18, or 21, the q15q22 deletion, or Turner syndrome, as well as other congenital anomalies, including scoliosis, macrognathia, encephalocele, spina bifida, and hypospadias [6].

Multifocal FMD is diagnosed based on the characteristic string-of-beads appearance observed in imaging studies [4]. These string-of-beads lesions are typically found in medium-sized arteries, such as the renal, iliac, carotid, and/or visceral arteries. In addition to the string-of-beads appearance, other abnormalities of medium-sized arteries may occur, including aneurysms, arterial tortuosity, and spontaneous dissections. Symptoms vary and likely depend on the affected arteries and can include headaches, pulsatile tinnitus, and abdominal complaints, as well as more severe manifestations like stroke or myocardial infarction in the case of spontaneous dissection [4]. Renovascular hypertension occurs in a subset of patients with renal artery FMD. Balloon angioplasty can be remarkably effective in lowering blood pressure, although this effect diminishes with age and is not seen in all patients. The reasons why some patients with renal artery multifocal FMD develop hypertension while others do not, why it has little to no effect on kidney function, and why responses to balloon angioplasty vary considerably, remain incompletely understood [9]. Similarly, the cause of the vascular abnormalities in multifocal FMD remains unknown, but atherosclerotic or inflammatory processes are considered unlikely. Extensive genetic studies have not identified any monogenic causes, but only polymorphisms that are associated with a slight increase in risk for FMD. Management of FMD is predominantly conservative, with periodic follow-up to detect new-onset hypertension and/or the development of aneurysms. In such cases, renal artery balloon angioplasty or (endo)vascular treatment of aneurysms may be considered [4].

At first glance, the co-occurrence of a horseshoe kidney and multifocal FMD in a single patient may seem coincidental. However, given the very low prevalence of both (with horseshoe kidney occurring in approximately 0.1-0.2% of the population and multifocal FMD being rare with an unknown prevalence) [4,6,8], the statistical probability of their co-occurrence is even lower. Considering that a horseshoe kidney is frequently associated with various congenital anomalies (e.g., chromosomal abnormalities, scoliosis, as previously discussed), this observation raises the possibility of a previously unrecognized congenital connection between FMD and a horseshoe kidney. Furthermore, one could also speculate that a mechanical factor may contribute to the development of renal artery FMD in a horseshoe kidney. Although highly speculative, it has been hypothesized that repeated microtrauma to the vascular wall could contribute to the development of the typical string-of-beads lesions as observed in multifocal FMD [10]. This may only occur in patients with pre-existing compromised arterial wall integrity (for example, those with polymorphisms that slightly increase the risk of FMD), which could explain increased vascular tortuosity and predisposition to dissections in those patients. In line with that hypothesis, one could speculate that a horseshoe kidney may increase mechanical stress on the arterial walls. The fusion of the kidneys restricts normal mediolateral movement within the retroperitoneal space, which may lead to repetitive craniocaudal movement of the relatively large, malrotated renal mass. This may cause renal artery microtrauma and thus contribute to the development of multifocal FMD. Further research could explore these hypotheses, particularly by assessing the prevalence of multifocal FMD and the biomechanical effects of the large, malrotated kidney mass in larger cohorts of patients with horseshoe kidneys.

## Conclusions

We report a rare case of multifocal renal artery FMD in a patient with a horseshoe kidney, a combination not previously described. Although this remains the only reported case, the co-occurrence of these two rare conditions raises the hypothesis of a potential association, possibly through shared embryologic mechanisms or altered biomechanical forces related to renal fusion and malrotation. From a research perspective, multicenter studies involving larger cohorts are warranted to assess the prevalence of FMD in patients with horseshoe kidneys and to investigate whether the biomechanical impact of the large, malrotated renal mass contributes to vascular remodeling or disease.
